# Anatomopathological aspects of osteoarticular lesions associated with *Rhodococcus equi* infection in two foals

**DOI:** 10.1007/s11259-026-11317-x

**Published:** 2026-07-20

**Authors:** Felipe Martins Pastor, Suzyane Oliveira Barros, Letícia Fernandes Campos, Acácia Eduarda de Jesus Nascimento, Gabriel Henrique Guimarães, Carlos Renato de Souza Guimarães Filho, Luis Ernesto Campos Torres, Pâmela Aparecida de Lima, Andressa Batista da Silveira Xavier, Rodrigo Otávio Silveira Silva, Roberto Maurício Carvalho Guedes, Rogéria Serakides, Roselene Ecco

**Affiliations:** 1 Department of Veterinary Clinic and Surgery, Veterinary School, Universidade Federal de Minas Gerais - UFMG, Avenida Antônio Carlos 6627, Belo Horizonte, Minas Gerais 31270-901 Brasil; 2 Department of Preventive Veterinary Medicine, Veterinary School, Universidade Federal de Minas Gerais - UFMG, Avenida Antônio Carlos 6627, Belo Horizonte, Minas Gerais 31270-901 Brasil

**Keywords:** Arthritis, Equine, Lameness, Osteomyelitis, Pyogranulomas, *Rhodococcus*

## Abstract

*Rhodococcus equi* is an important bacterium that affects foals up to six months of age, especially those that are immunocompromised. The main manifestation is pyogranulomatous pneumonia; nevertheless, extrapulmonary lesions have not only been reported, but are not uncommon, including arthritis and osteomyelitis. Despite the widespread occurrence of rhodococcosis, the macro- and microscopic lesions in the musculoskeletal system are still poorly understood, and a detailed study is needed to better understand the pathogenic potential of the organism. The aim of this report is to present the anatomopathological aspects of bone and joint lesions associated with *Rhodococcus equi* in two foals. The first case was a two-month-old male Quarter Horse foal with suspected cervical trauma. This horse was admitted in lateral recumbency, semi-comatose, and with atlantoaxial region enlarged. Necropsy revealed atlantooccipital arthritis with pyogranulomatous osteomyelitis, resulting in pathologic fractures of the atlas. There was also pyogranulomatous pachymeningitis leading to compressive myelopathy. In the second case, a two-month-old Brazilian Sport Horse foal presented with lameness of the left pelvic limb and enlargement of the left femorotibial and femoropatellar and right scapulohumeral joints. Necropsy revealed femorotibial and femoropatellar arthritis and purulent osteomyelitis in the femur, as well as extensive necrosis and bone rarefaction with focal detachment of the epiphyseal plate. Both animals had pyogranulomatous pneumonia and lymphadenitis with extracellular and intracytoplasmic gram-positive coccobacilli in neutrophils and macrophages. In the first case, *R. equi* was isolated and confirmed by MALDI-TOF. *R. equi* infection should be considered in the differential diagnosis of osteoarticular lesions in foals.

## Introduction

*Rhodococcus equi* is a gram-positive, aerobic, facultative intracellular coccobacillus, partially acid resistant, and is commonly found in soil and decomposing organic matter. Like *Corynebacterium*, *Mycobacterium* and *Nocardia*, the genus *Rhodococcus* belongs to the Mycolata family of actinomycetes (Ruocco et al. 2020; Pal et al. 2023; Jhandai et al. 2024). The microorganism was initially described as *Corynebacterium equi* based on its morphological and phenotypic characteristics (Magnusson 1923). Subsequently, it was reclassified into the genus *Rhodococcus* (Goodfellow and Alderson 1977). Based on taxonomic priority rules, the nomenclature *Rhodococcus hoagii* was proposed (Kämpfer et al. 2014); however, given the longstanding and widespread use in medical and veterinary literature as well as in diagnostic practice, *R. equi* remains the preferred nomenclature (Vázquez-Boland et al. 2020).

*R. equi* has a cell wall rich in mycolic acid (Vázquez-Boland et al. 2013; Pal et al. 2023) and a polysaccharide capsule that allows immune evasion and prolonged survival in the environment (Pal et al. 2023). Pathogenic strains of *R. equi* contain virulence-associated plasmid (Vap) proteins, which are essential for survival and proliferation within macrophages (Vázquez-Boland et al. 2013; Sanz 2023). Strains commonly isolated from horses have the VapA protein (Prescott 1991; Takai et al. 1991a, b; Tkachuk-Saad and Prescott 1991; Hondalus 1997; Benoit et al. 2001), while human and other animal isolates tend to encode the VapB variant (Takai et al. 2000; Byrne et al. 2001; Oldfield et al. 2004).

The disease is most prevalent in young horses, especially foals between one and six months of age (Morresey et al. 2011; Ruocco et al. 2020; Sanz 2023). The high susceptibility of foals in this age group is attributed to the decline in passive immunity, the immaturity of active immunity, and the high exposure to the pathogen in contaminated environments such as paddocks (Morresey et al. 2011). The primary route of infection is aerogenous, and after internalization by alveolar macrophages and intracellular proliferation, the bacterium induces a strong inflammatory response that can culminate in severe pyogranulomatous pneumonia (Ruocco et al. 2020; Sanz 2023).

Although *R. equi* is more commonly associated with infections in young animals, the disease can occur in adult horses when they have compromised immune systems (Clark-Price et al. 2003; Sanz 2023). Infection in pigs, cattle, goats, cats and dogs is less common (Pate et al. 2004; Bryan et al. 2017; Jhandai et al. 2024). In addition, *R. equi* can cause serious infections in humans, especially in immunocompromised individuals such as HIV/AIDS patients, transplant recipients, or those treated with immunosuppressants (Torres-Tortosa et al. 2003; Kilcoyne et al. 2014; Shah et al. 2020). The main routes of infection in humans are aerogenous or oral (Weinstock and Brown 2002; Shah et al. 2020).

In severe and septic conditions, the bacteria can spread to extrapulmonary tissues, resulting in nodal, intestinal, and osteoarticular lesions (Janicek et al. 2006; Morresey et al. 2011). Arthritis and osteomyelitis have occasionally been reported in association with infection with *R. equi* (Zink et al. 1986; Reuss et al. 2009; Oliveira et al. 2019; Ruocco et al. 2020; Labordère et al. 2022). Despite this, more in-depth descriptions of the macroscopic and microscopic lesions are important to improve the understanding of the pathogenic potential of the bacterium. The aim of this report is to present the anatomopathological aspects of bone and joint lesions associated with *Rhodococcus equi* in two foals.

## Case report

The owners provided informed consent for their foals’ data to be used in this study.

### Case 1

#### Clinical history

A 2-month-old male Quarter Horse foal was referred for clinical care with suspected cervical trauma three days ago, when clinical signs of difficulty walking and standing were observed. The animal was admitted to the veterinary hospital in semi-comatose condition, in lateral recumbency and with an enlargement of the atlantoaxial joint. Neurological examination revealed dysfunction of cranial nerves (optic, oculomotor, trigeminal and vestibulocochlear), decreased cervicofacial reflexes and spastic paralysis of thoracic and pelvic limbs. Supportive treatment was initiated with dimethyl sulfoxide, dexamethasone, and flunixin megluminate, as well as parenteral fluid therapy with 3% hypertonic saline. Despite treatment, general condition worsened and the owner decided for euthanasia, as surgical intervention was not possible.

#### Post-mortem findings

**Fig. 1:**
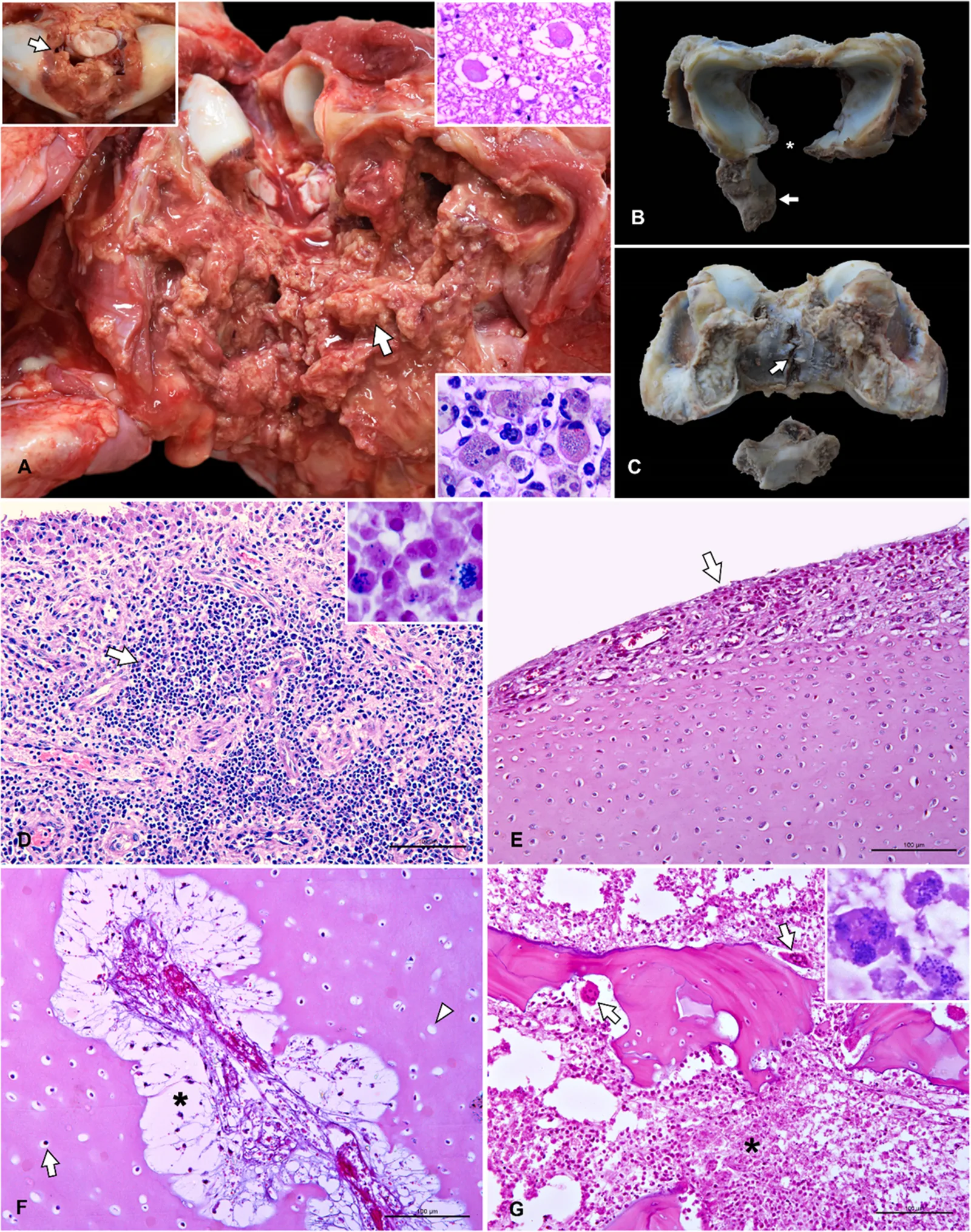
Case 1, Quarter Horse foal, two-month-old. (**A**) Atlantooccipital joint filled with a large amount of thick, cloudy and reddish-yellow exudate (arrow). Upper left detail: Caudal view of the condyles of the occipital bone with exudate filling the foramen magnum and surrounding the spinal cord (arrow). Upper right detail: Spinal cord corresponding to the atlas with loss of axons and axonal spheroids. Lower right detail: Retropharyngeal lymph node with necrotic and pyogranulomatous lymphadenitis associated with intralesional coccobacilli visible by HE staining. (**B**) Atlas, cranial view, complete fracture of the ventral arch (asterisk), with detachment of a bone fragment of approximately 4 cm (arrow). (**C**) Atlas, ventral view, incomplete fracture of the dorsal arch (arrow). (**D**) Thick synovial membrane, with marked lymphohistiocytic inflammatory infiltrate, with multinucleated giant cells and neutrophils (arrow). HE, bar: 100 μm. Detail: Gram-positive coccobacilli in the cytoplasm of macrophages. (**E**) Articular cartilage covered by highly vascularized fibrous connective tissue (pannus), interspersed with neutrophils, macrophages and cell debris (arrow). HE, bar: 100 μm. (**F**) Articular cartilage with pyknotic chondrocytes (arrow), empty lacunae (arrowhead) and focus of chondronecrosis (asterisk). HE, bar: 100 μm. (**G**) Fragmented and disconnected bone trabeculae, with osteoclasts adhered in Howship’s lacunae (arrows); bone marrow filled with inflammatory cells and cellular debris (asterisk). HE, bar: 100 μm. Detail: Gram-positive coccobacilli in the cytoplasm of macrophages

Grossly, there was moderate enlargement of the atlantooccipital and atlantoaxial joints. The joint space was filled with a large amount of viscous, cloudy, red-yellow fluid containing suspended solid particles (Fig. 1a). The joint capsule was thick and red, covered with yellow filaments and clumps. The exudate infiltrated the spinal canal and filled the subdural space up to the brain. The dura mater in the brainstem region was thick, yellow, and covered with friable fibrin filaments. The atlas had reduced bone strength and there were two complete fractures in the ventral arch with detachment of approximately 4 cm of bone. There was also one incomplete fracture in the dorsal arch (Fig. 1b, c). The retropharyngeal and tracheobronchial lymph nodes were markedly enlarged, and the parenchyma was replaced by pyogranulomatous inflammation. There were no hemorrhages or muscle tears in the cervical region, which would indicate changes of traumatic origin.

In the lung, there were two nodules, poorly demarcated, elevated, pale yellow, about 4.5 cm in diameter, firm, and surrounded by a dark red halo; on section, the nodules consisted of pyogranulomatous inflammation.

#### Histopathological findings

The synovial membrane of the atlantooccipital joint showed a proliferation of synoviocytes with papillary processes and a marked inflammatory infiltrate, predominantly lymphohistiocytic, with occasional multinucleated Langhans giant cells, neutrophils, and cellular debris. Large numbers of weakly basophilic bacilli were found in the cytoplasm of macrophages (Fig. 1d). In the atlas, the articular cartilage was covered by highly vascularized fibrous connective tissue (pannus) interspersed with neutrophils, foamy macrophages, and cellular debris (Fig. 1e). The cartilage was irregular, with fibrillation in the superficial layer, and contained undifferentiated chondrocytes, sometimes pyknotic, and empty lacunae. There were foci of absence of chondroid matrix, filled with fibrin, neutrophils and red blood cells, which sometimes coalesced and formed fissures that dissected fragments of cartilage (chondronecrosis) (Fig. 1f). Bone trabeculae were fragmented and disconnected, with moderate numbers of osteoclasts lodged in Howship’s lacunae. There were large numbers of neutrophils, foamy macrophages, and fibrin at the edges of the fracture. The cortical bone was thin and also contained osteoclasts adhering to Howship’s lacunae. The periosteum was thick, highly fibrous, and interspersed with neutrophils and foamy epithelioid macrophages. Bone marrow was intensely filled with neutrophils and foamy, epithelioid macrophages, with numerous cellular debris (Fig. 1g). Gram’s histochemical stain showed a myriad of blue-violet coccobacilli, 2–3 μm in diameter in the cytoplasm of neutrophils and macrophages, and extracellular. The anatomopathological diagnosis was confirmed as pyogranulomatous and necrotizing arthritis and osteomyelitis with pathological fractures, associated with gram-positive coccobacilli. In the dura mater, the inflammatory infiltrate was composed of neutrophils, epithelioid macrophages and multinucleated Langhans giant cells, characterizing marked multifocal to coalescing pyogranulomatous pachymeningitis. In the spinal cord corresponding to the atlas and axis there was multifocal compressive myelopathy, with loss of axons, digestion chambers, axonal spheroids and marginal chromatolysis (Fig. 1a, inset).

The retropharyngeal and tracheobronchial lymph nodes had necrotic and pyogranulomatous lymphadenitis with intralesional coccobacilli on HE and Gram stains, with replacement of most of the normal parenchyma (Fig. 1a, inset). The pulmonary nodules were characterized as pyogranulomas associated with intralesional gram-positive coccobacilli.

#### Complementary tests

Synovial fluid and pulmonary pyogranuloma samples were submitted to bacterial culture on BHI (Brain Heart Infusion, Difco, USA) agar supplemented with 5% sheep blood. Growth of colonies compatible with *Rhodococcus equi* was identified, confirmed by Matrix-Assisted Laser Desorption/Ionization Time-Of-Flight Mass Spectrometry (MALDI-TOF, Bruker Daltonics, Bremen, Germany), as previously described (Assis et al. 2017).

### Case 2

#### Clinical history

A 2-month-old male Brazilian Sport Horse foal was referred to the clinic with lameness of the left pelvic limb and increased volume in the left femorotibial and femoropatellar and right scapulohumeral joints. The foal was obtunded and presented with diarrhea, recurrent fever and a superficial respiratory pattern. There was marked leukocytosis (28.500 cells/µl; reference: 5.200–13.900) due to neutrophilia with right shift (26.505 cells/µl; reference: 2.200–8.500), as well as elevated serum fibrinogen (1.200 mg/dL; reference: 100–400). Treatment with antibiotics and anti-inflammatories was initiated, but there was no clinical improvement. The medical records did not include information about medications, doses, or the duration of treatment. Given the poor prognosis, the owner opted for euthanasia.

#### Post-mortem findings

**Fig. 2:**
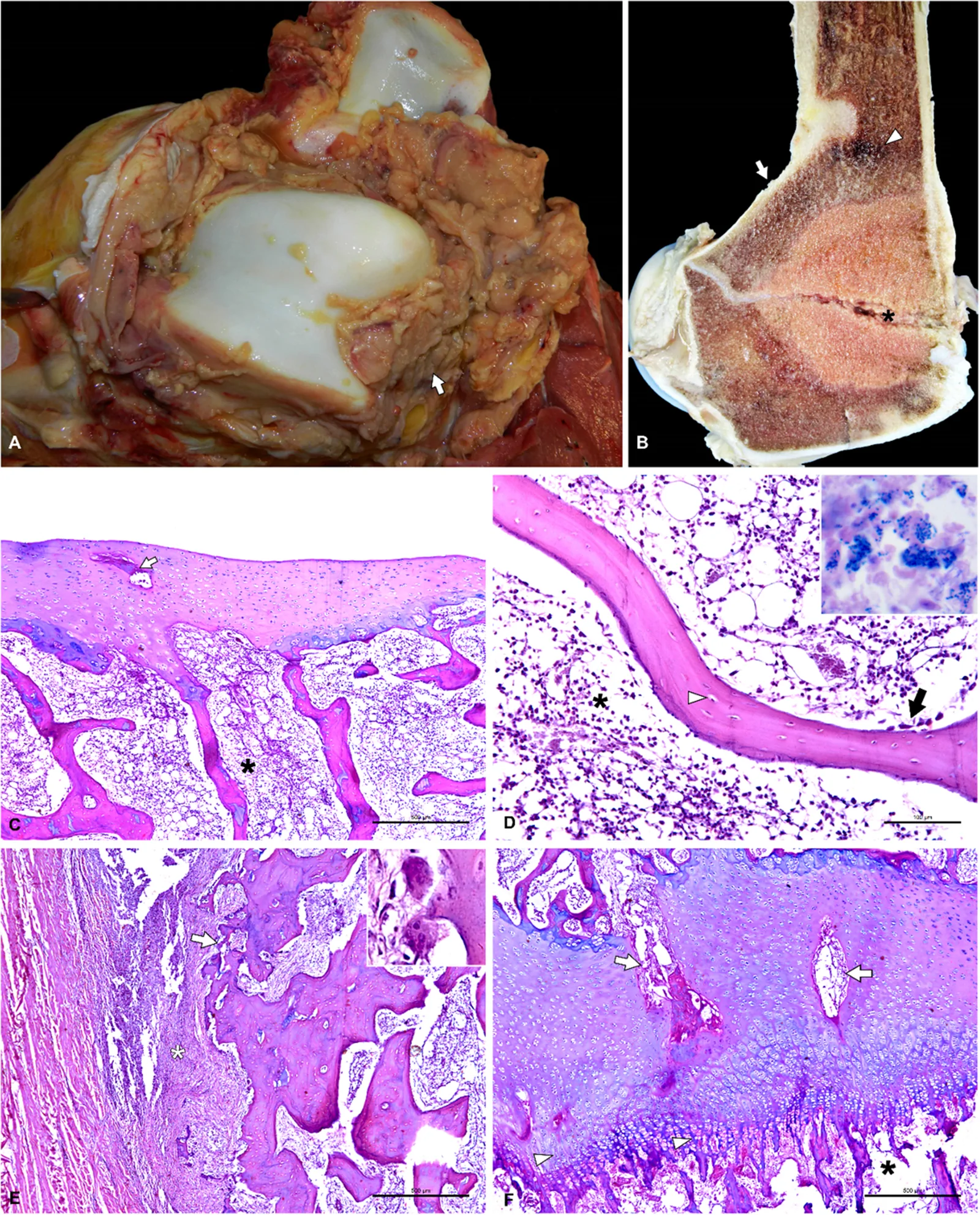
Case 2, Brazilian Sport Horse foal, two-month-old. (**A**) Femoro -patellar joint with a large amount of yellow, viscous material, with yellow, friable, lumpy filaments in the joint space and adhered to the synovium (arrow). (**B**) Longitudinal section of the femur, epiphysis and metaphysis adjacent to the epiphyseal plate with a focally extensive, pinkish area with disintegration of the epiphyseal plate (asterisk); the bone marrow is tan with a focal area of hemorrhage (arrowhead); thin, irregular cortical bone (arrow). (**C**) Irregular and thin articular cartilage, with focus of chondronecrosis (arrow); bone marrow of the femoral epiphysis replaced by inflammatory cells (asterisk). HE, bar: 500 μm. (**D**) Thin bone trabeculae, bordered by a single layer of flattened osteoblasts (arrow), with osteocytes housed in widened lacunae (arrowhead); bone marrow with neutrophils and macrophages (asterisk). HE, bar: 100 μm. Detail: Gram-positive intra and extracellular coccobacilli. (**E**) Irregular and discontinuous cortical bone (arrow), with intense periosteal reaction and neutrophilic inflammatory infiltrate (asterisk). HE, bar: 500 μm. Detail: osteoclasts adhered in Howship’s lacunae. (**F**) Irregular epiphyseal plate, with areas of chondronecrosis (arrows), marked reduction in the number of proliferative and hypertrophic chondrocytes (arrowheads), and detachment of the epiphyseal plate from the metaphyseal trabeculae (asterisk). HE, bar: 500 μm

The medial side of the left pelvic limb, in the region of the femorotibial joint, was markedly enlarged. The subcutaneous tissue at this location and muscular fascia contained a large amount of light yellowish viscous fluid (phlegmon). There was marked thickening of the joint capsule and a large amount of yellow and viscous material with friable, lumpy filaments, sometimes adherent to the cartilage and synovium; the articular cartilage had an irregular surface (Fig. 2a). On longitudinal section, the femur showed a focally extensive, pinkish area in the epiphysis and metaphysis adjacent to the epiphyseal plate with disintegration of the growth plate. The marrow was tan, with a focal area of hemorrhage, and the cortical bone was irregular and thin (Fig. 2b). Similar findings were observed in the scapulohumeral joint.

In the large colon, dozens of yellowish-white multifocal nodules ranging in size from 0.5 to 2.0 cm were seen in the serosa. On sectioning, the nodules were composed of caseous-purulent material and extended to the mucosa, which was thick and irregular with multiple ulcers between 2.0 and 10.0 cm in diameter. Multifocal, poorly-defined nodules, approximately 1.0 to 9.0 cm in diameter, pale yellow and firm, were present in the lungs. On sectioning, there was an intense amount of white, viscous exudate with friable clots. Peripheral, tracheobronchial, mesenteric, cecal, and colonic lymph nodes were enlarged and there was no cortico-medullary distinction with parenchyma replaced by yellowish and viscous exudate.

#### Histopathological findings

The articular cartilage of the femur was irregular, thin, with multiple areas of fibrillation and empty chondrocyte lacunae (chondronecrosis) (Fig. 2c). The epiphyseal and metaphyseal trabeculae were rarefied, thin and disconnected, covered by rare flattened osteoblasts; the osteocytes were small and sometimes there were empty with enlarged lacunae (osteocytic osteolysis) (Fig. 2d). The cortical bone was irregular, sometimes trabecular in appearance, and contained multiple osteoclasts lodged in Howship’s lacunae. In addition, there was an intense periosteal reaction with new bone formation and a neutrophilic inflammatory infiltrate (Fig. 2e). The bone marrow contained intact and degenerated neutrophils, a moderate number of foamy macrophages, and a large amount of fibrin filaments. Gram’s histochemical staining showed an abundance of blue-violet coccobacilli (approximately 2–3 μm) in the cytoplasm of neutrophils and macrophages, and extracellular. The epiphyseal plate was irregular, with markedly thin areas along with marked reduction in the number of chondrocytes in the proliferative and hypertrophic zones. Extensive foci of separation of the epiphyseal plate from the bone metaphysis were also present (Fig. 2f). The findings led to the diagnosis of necropurulent arthritis and osteomyelitis with intense bone rarefaction, necrosis and focal detachment of the epiphyseal plate, associated with gram-positive coccobacilli.

In the large colon, there was marked multifocal transmural pyogranulomatous ulcerative colitis associated with pyogranulomatous lymphadenitis. The lungs had marked multifocal to coalescent pyogranulomatous pneumonia. The lymph nodes had similar changes, with loss of parenchyma and tissue replacement by multiple pyogranulomas. The intestinal, pulmonary and lymph node lesions were also associated with gram-positive coccobacilli extracellularly, and in the cytoplasm of macrophages and multinucleated giant cells. Confirmatory tests, including bacteriology culture and immunohistochemistry, were not performed in this second case.

## Discussion

This report describes two different presentations of arthritis and osteomyelitis associated with *Rhodococcus equi* in foals. In the first case, the inflammatory changes were localized in the retropharyngeal lymph nodes and in the atlanto-occipital and atlanto-axial joints, culminating in pathologic fractures of the atlas with consequent compressive myelopathy and neurologic manifestations. The initial clinical suspicion of cervical trauma was ruled out because the fractures in the atlas were secondary to the extensive osteomyelitis of the vertebra and were therefore considered pathologic. In the second case, the changes were localized in joints of the appendicular skeleton, such as the femorotibial, femoropatellar and scapulohumeral joints. There was a rarefactive osteomyelitis with focal detachment of the epiphyseal plate, which could lead to epiphysiolysis if the disease progressed. In both cases, there was an abundance of gram-positive coccobacilli in the cytoplasm of macrophages and neutrophils, and extracellularly.

Another similarity is that both animals had pulmonary pyogranulomas, a classic lesion of *R. equi* infection. The pulmonary manifestation is the most commonly observed in foals, either as a single location or associated with extrapulmonary manifestations. In a clinical and anatomopathologic study, pulmonary pyogranulomas were observed in 95% of the animals examined (Oliveira et al. 2019). Other manifestations observed in this report, such as lymphadenitis and ulcerative colitis, are also referred to as common lesions, and were diagnosed in 50% and 25% of affected foals, respectively (Zink et al. 1986; Oliveira et al. 2019).

Osteoarticular lesions associated with *R. equi* are considered difficult to diagnose and treat (Hepworth-Warren et al. 2015). Bacteremia is the primary pathway for the pathogen to reach the bones and joints as a common consequence of pyogranulomatous pneumonia (Glass and Watts 2017). The organism colonizes sites such as the marrow sinusoids of the epiphysis and metaphysis, which have lower blood flow and pressure (Kelleher and MacDonald 2007). Other routes of bone infection include contiguous spread from septic arthritis or direct entry through skin lesions (Hance 1998). There may be clinical manifestations of arthritis, osteomyelitis and subcutaneous abscesses, and in these cases the prognosis is considered poor (Desjardins and Vachon 1990; Chaffin et al. 1995; Paradis 1997).

In addition to *R. equi*, other Mycolata group organisms have been identified in equine osteoarticular lesions, but reports are scarce. *Corynebacterium pseudotuberculosis* has already been associated with polyarthritis and osteomyelitis (Nogradi et al. 2012). *Mycobacterium* organisms have been identified in cases of granulomatous arthritis and synovitis (*M. avium*) (Hewes et al. 2005), and granulomatous osteomyelitis in ribs, sternum and cervical and thoracic vertebrae (*Mycobacterium bovis*) (Kelly et al. 1972).

In both cases reported in the present study, arthritis was an important finding. In other studies, the incidence of arthritis ranged from 30 to 34% (Reuss et al. 2009; Oliveira et al. 2019). The most common sites are the appendicular joints, such as: scapulohumeral, humero-radio-ulnar, carpal, metacarpophalangeal, interphalangeal, coxofemoral, femorotibial, femoropatellar, tibiotarsal, tarsal, and metatarsophalangeal joints. (Reuss et al. 2009; Oliveira et al. 2019; Ruocco et al. 2020). A predominantly lymphohistiocytic inflammatory infiltrate with Langhans cells and neutrophils was observed in the synovia of these cases. Lymphoplasmacytic inflammation is reported to be the most common, followed by neutrophilic and pyogranulomatous inflammation. In the lymphoplasmacytic arthritis, usually immune-mediated (Sweeney et al. 1987; Giguère and Prescott 1997), identification of the agent is difficult, either by bacterial culture or molecular tests such as immunohistochemistry (Oliveira et al. 2019). In the first case of this report, the pathogen was easily identified by both histochemical Gram staining and bacterial culture. In the second case, although the lack of bacterial isolation and immunohistochemical confirmation represents a limitation, it did not hinder the comprehensive interpretation of lesions or the establishment of a pathological diagnosis.

Osteomyelitis is reported as less frequent than arthritis in cases of *R. equi* infection, and can affect around 12% of foals with extrapulmonary manifestations (Reuss et al. 2009). Lesions can be found in the vertebrae (Oliveira et al. 2019), but the involvement of the appendicular skeleton bones has already been described (Collatos et al. 1990; Desjardins and Vachon 1990; Firth et al. 1993; Paradis 1997; Clark-Price et al. 2003; Loesch et al. 2003; Kelleher and MacDonald 2007; Reuss et al. 2009; Ruocco et al. 2020; Labordère et al. 2022). In both cases of this report, osteomyelitis was associated with arthritis. However, other studies have reported that the bone lesion occurred independently of the joint change (Desjardins and Vachon 1990; Firth et al. 1993; Paradis 1997; Clark-Price et al. 2003).

In the first case of this report, the osteomyelitis was pyogranulomatous, similar to the morphologic pattern observed in the lungs. In the cases available in the literature where the microscopic description of the bone and lung lesions is included, both were classified as pyogranulomatous (Morresey et al. 2011; Oliveira et al. 2019). However, in the second case, the osteomyelitis was purulent, whereas in the lungs and lymph nodes, the inflammatory infiltrate was predominantly pyogranulomatous. Our hypothesis is that the difference in the morphology of the inflammatory infiltrate may be related to the late arrival of the pathogen in the bone, since the lower vascularization of cartilage and bone compared to other organs may contribute to the delay of bacterial colonization in infections of hematogenous origin.

Vertebral osteomyelitis is an important lesion because of the extension of the inflammatory process to the central nervous system. The accumulation of exudate can also cause compressive myelopathy, which may manifest with neurological signs depending on the region involved. In the first case of this report, multifocal myelopathy was observed, which was possibly attributed to the intermittent compression of the spinal cord by the bone fragments and the purulent exudate during movement of the joint. There is a single report of involvement of the atlantooccipital region with neurological manifestations (Morresey et al. 2011). The case involved a 3-month-old Thoroughbred foal with ataxia, neck extension and local pain, as well as hypermetria and hoof dragging when walking. Magnetic resonance imaging of the joint revealed alterations compatible with arthritis, osteomyelitis and neural compression. The foal was euthanized and necropsy revealed extensive arthritis with pyogranulomatous osteomyelitis of the occipital bone and brainstem compression from pyogranulomatous meningitis (Morresey et al. 2011). Neurological signs have also been described in other cases of osteomyelitis in foals with involvement of cervical, thoracic or lumbar vertebrae (Giguére and Lavoie 1994; Olchowy 1994; Oliveira et al. 2019). In another study, a foal with pyogranulomatous osteomyelitis of the atlas did not show any neurological signs or changes in the central nervous system (Oliveira et al. 2019).

Regarding other animal species, there are few reports of osteoarticular lesions associated with *R. equi*. In dogs, only one case of pyogranulomatous osteomyelitis of the scapula and humerus has been reported in a 3-month-old Basenji female dog (Cantor et al. 1998). In goats, osteomyelitis has already been described in the tibia (Haanen et al. 2020), humerus (Stranahan et al. 2018), atlas (Kabongo et al. 2005), occipital, thoracic vertebrae (Carrigan et al. 1988) and lumbar vertebrae (Stranahan et al. 2018), with spinal cord compression in two cases (Carrigan et al. 1988; Stranahan et al. 2018). In all cases described in goats or dogs, aetiologic diagnosis was only established after necropsy, and the attempted therapy was apparently ineffective, with no clinical improvement.

In humans, cases of arthritis (Broughton et al. 1981; Verville et al. 1994; Athavale et al. 2024) and osteomyelitis have been described (Broughton et al. 1981; Novak et al. 1988; Antinori et al. 1992; Scott et al. 1995; Fischer et al. 1998; Sistla et al. 2009; Rallis et al. 2012; Tasnim et al. 2023), in association with *R. equi* (Athavale et al. 2024), or *R. erythropolis* (Roy et al. 2009). The treatment described, which was successful in most cases, included macrolides (azithromycin, clarithromycin or erythromycin) (Fischer et al. 1998; Sistla et al. 2009; Tasnim et al. 2023; Athavale et al. 2024), rifampicins (rifabutin or rifampin) (Fischer et al. 1998; Tasnim et al. 2023), carbapenems (imipenem or meropenem) (Rallis et al. 2012; Athavale et al. 2024), glycopeptides (vancomycin) (Tasnim et al. 2023; Athavale et al. 2024), oxazolidinones (linezolid) (Rallis et al. 2012), and quinolones (ciprofloxacin) (Roy et al. 2009). Most of the cases was described in immunocompromised patients (Novak et al. 1988; Fischer et al. 1998; Rallis et al. 2012; Tasnim et al. 2023) or elderly persons (Athavale et al. 2024). However, there were reports of osteoarticular lesions in immunocompetent people (Verville et al. 1994; Roy et al. 2009; Sistla et al. 2009).

In horses, antimicrobial treatment of *R. equi* is difficult because it is a facultative intracellular organism. Penetration of antibiotics into the intracellular environment is limited, and sites such as bones and joints are poorly vascularized compared to other tissues (Burton et al. 2015; Fenton and Buckley 2015; Glass and Watts 2017). Another limiting factor in the treatment of infection is the high resistance of the organism due to the indiscriminate use of antimicrobials (Burton et al. 2013, 2015) which include drugs such as rifampins and macrolides (Giguére et al. 2004; Giguère et al. 2010).

In only two cases of osteoarticular lesions associated with *R. equi* consulted in the literature, the treatment was successful and there was complete recovery. Kelleher and MacDonald (2007) described the case of a two-month-old Quarter Horse foal with osteomyelitis of the humerus. Treatment included surgical debridement and washing the lesion with enrofloxacin solution. Initial therapy consisted of penicillin G procaine, gentamicin, and flunixin megluminate; later the protocol was changed to clarithromycin and rifampicin due to pure growth of *R. equi* from the affected site, with complete recovery after 12 weeks. Clark-Price et al. (2003) described the case of a two-year-old Quarter Horse mare diagnosed with osteomyelitis of the pubic symphysis. Treatment consisted of surgical debridement of the lesion and antibiotic therapy including erythromycin and rifampicin. The lesion healed completely after four months and the mare was gradually reintroduced to exercise. Although success in treating osteoarticular lesions in horses is considered limited, it is important to note that early diagnosis and initiation of appropriate therapeutic procedures, such as removal of necrotic tissue and antibiotic therapy, are essential to ensure resolution of the infection.

## Conclusion

In this study, pyogranulomatous/necropurulent arthritis and osteomyelitis, with bone rarefaction and pathological fractures demonstrated the pathogenic potential of *R. equi* in the locomotor system. In the case with vertebral involvement, the lesions developed pachymeningitis and compressive myelopathy with consequent neurological clinical manifestations. In both cases, the pathological diagnosis was established based on necropsy and complementary tests such as histopathology and histochemical stains. Finally, *R. equi* infection should always be considered in the differential diagnosis of osteoarticular lesions in foals with clinical manifestation of articular and bone changes.

## Data Availability

No datasets were generated or analysed during the current study.
